# Proteomic results indicate inhibition of complement cascades is one of the mechanisms underlying the protective effects of Tongxinluo against myocardial infarction

**DOI:** 10.3389/fphar.2026.1866207

**Published:** 2026-07-10

**Authors:** Ziqin Zhou, Ce Zhang, Yao Jiang, Yuejin Yang, Guihao Chen, Qi Pan, Jimei Chen

**Affiliations:** 1 State Key Laboratory of Cardiovascular Disease, National Center for Cardiovascular Diseases, Fuwai Hospital, Chinese Academy of Medical Sciences and Peking Union Medical College, Beijing, China; 2 Guangdong Cardiovascular Institute, Guangdong Provincial People’s Hospital (Guangdong Academy of Medical Sciences), Southern Medical University, Guangdong, China

**Keywords:** acute myocardial infarction, C1qA, cardioprotection, complement cascade, proteomics, tongxinluo

## Abstract

**Objective:**

Tongxinluo (TXL), a traditional Chinese medicine, has demonstrated cardioprotective effects in acute myocardial infarction (AMI), yet its impact on myocardial protein profiles remains largely unexplored. This study aimed to elucidate the proteomic mechanisms underlying the therapeutic effects of TXL in AMI.

**Methods:**

AMI was induced via left anterior descending artery ligation in C57BL/6J mice. TXL was administered by oral gavage during the acute phase. Myocardial tissues and serum were subjected to 4D label-free quantitative proteomics, Western blotting, ELISA, immunofluorescence, TUNEL staining, and molecular docking analyses. Cardiac function was evaluated by echocardiography and fibrosis was quantified by Sirius red staining.

**Results:**

Proteomic analysis identified 1,061 differentially expressed proteins between TXL- and vehicle-treated AMI mice, with KEGG enrichment highlighting significant downregulation of complement and coagulation cascades. Key complement components C1qa and C1s were markedly upregulated post-AMI but significantly suppressed by TXL. Molecular docking suggested potential interactions between multiple TXL bioactive compounds and C1qa/C1s. TXL treatment reduced complement activation (serum C3a), inhibited cardiomyocyte apoptosis (Bax/Bcl-2, TUNEL), improved left ventricular ejection fraction, and attenuated fibrosis. Notably, TXL monotherapy outperformed C1 inhibitor (C1i) alone, while combining C1i with TXL conferred no additional benefit, indicating that C1 complement inhibition is an intrinsic component of TXL’s mechanism.

**Conclusion:**

Modulation of C1-associated complement activation may be one of the mechanisms through which TXL protects against AMI, providing novel proteomic-level insights into the cardioprotective actions of this traditional Chinese medicine.

## 1 Introduction

Acute myocardial infarction (AMI) represents a significant global health threat, associated with substantial mortality (Lindahl and Mills, 2023). Despite the advancements in reperfusion therapy and optimal medical management, patients with AMI continue to face elevated risks of both in-hospital mortality and recurrent cardiovascular events (Figtree et al., 2021; Hillerson et al., 2022; Xu et al., 2021). Complement activation has been considered an important factor for myocardial ischemic injury. Initiated by molecules such as component 1 (C1) and lectin, the cascade activation of C3 and C5 occurs, leading to the formation of the membrane attack complex (MAC) and resulting in tissue damage (Garred et al., 2021). Therapeutic efficacy in experimental AMI models has been reported for a series of complement-targeting agents, including cobra venom factor (CVF), soluble complement receptor 1, C1-esterase inhibitor (C1i), FUT-175, etc (Garred et al., 2021; Emmens et al., 2017).

Tongxinluo (TXL), a traditional Chinese medicinal formulation, consists of a complex mixture of powders and extracts derived from various plant and insect components (Zhang et al., 2009). TXL was first assessed and approved for clinical application in China in 1996 for the management of angina pectoris and ischemic stroke. Preclinical studies have revealed that TXL pretreatment enabled the reduction of endothelial cell apoptosis by inducing autophagy, and mitigated hypoxia/reoxygenation-induced injury in cardiomyocytes via activating endothelial nitric oxide synthase (eNOS) and promoting angiogenesis (Li et al., 2010; Chen et al., 2020). In a randomized, double-blind, placebo-controlled clinical trial (CTS-AMI) of 3777 patients with ST-elevated AMI, we found that 12-week oral administration of TXL significantly reduced the primary endpoint of 30-day major adverse cardiovascular and cerebrovascular events (3.4% vs. 5.2% in the placebo group), with a significant reduction in cardiac death (3.0% vs. 4.2%). These benefits persisted within 1 year, with no significant difference in major bleeding (Yang et al., 2023). However, there is a lack of investigation into the changes in myocardial protein profiles under TXL treatment, which hinders a comprehensive understanding of the mechanisms by which this composite formulation exerts its anti-myocardial ischemia effects.

Herein, TXL was administrated to AMI mice and hearts were harvested. Proteomic analysis was performed and the complement system including C1s and C1qa was identified as one of the mechanisms underlying the cardioprotective effects of TXL. The proteomics dataset included a database-annotated C1s1 entry, while subsequent experimental validation was performed at the C1s protein level. These findings provide novel insights into understanding the role of traditional Chinese medicines (TCM) in treating and managing AMI.

## 2 Methods

### 2.1 Reagents and antibodies

The TXL ultrafine powder was purchased from Shijiazhuang Yiling Pharmaceutical Co. Ltd. (China) and dissolved in normal saline (NS) before injection. C1 inhibitor (C1i, Serpin G1; Cat No. HY-P71297) was obtained from MedChemExpress (United States) and dissolved in NS. RIPA lysis (Cat No. R0010) was purchased from Solarbio (China). Tribromoethanol was obtained from Sigma-Aldrich (United States). Rabbit anti-C1s (Cat No. A6878), anti-C1qa (Cat No. A24519), anti-Bcl-2 (Cat No. A19693), anti-Bax (Cat No. A19684), and anti-GAPDH (Cat No. A19056) antibodies used for Western blot (WB) and immunofluorescent staining were purchased from Abclonal (China). HRP-conjugated (Cat No. SA00001-2) and Fluorescein (FITC)–conjugated Goat Anti-Rabbit IgG (H + L) (Cat No. SA00003-2) was purchased from Proteintech (China). BCA protein assay (Cat No. P0010), 5× SDS-polyacrylamide (SDS-PAGE) sample Loading Buffer (Cat No. P0015), Antifade Mounting Medium with 4′,6-diamidino-2-phenylindole (DAPI; Cat No. P0131) and Terminal deoxynucleotidyl transferase dUTP nick end labeling (TUNEL) kit (Cat No. C1088) were obtained from Beyotime (China). Sirius Red staining kit (Cat No. BP-DL029) was obtained from Sbjbio (China). Mouse C3a Enzyme-linked immunosorbent assay (ELISA) kit (Cat No. E-EL-M0337) was obtained from Elabscience (China).

### 2.2 Animal experiments

All animal studies were approved by The Animal Care and Use Committee of Fuwai Hospital, Peking Union Medical College of Medicine (FW-2018-0010), in accordance with Public Health Service guidelines. Eight-week-old male wildtype C57BL/6J mice (20–24 g, Charles River) were randomly assigned to different experimental groups using a computer-generated random number sequence before surgery. The randomization sequence was generated by an investigator who was not involved in surgery, treatment administration, outcome assessment, or data analysis. After anesthetization intraperitoneal injection of 2% tribromoethanol (10 μL/g body weight) before the surgery procedure, ligation of left anterior descending coronary artery (LAD) was performed; while the sham group underwent a similar surgical procedure without ligation. No release of the ligature or reperfusion procedure was performed in this model. For proteomic analysis, there were three groups: 1) Sham, 2) AMI + NS, 3) AMI + TXL,with n = 6 mice per group included in the final proteomic analysis. For treatment study, there were five groups: 1) Sham, 2) AMI + NS, 3) AMI + C1i alone, 4) AMI + TXL alone, 5) AMI + TXL + C1i, with n = 6 mice per group included in the final analysis. Treatments were conducted during the first 3 days post-AMI (acute stage). TXL was given by gavage (0.75 g/kg bodyweight daily) (Wang et al., 2022). C1i was administrated intravenously (0.4 IU/g bodyweight) (Lu et al., 2013). NS was used as the vehicle. At day 3, 7, and 21 post-AMI, echocardiography was performed; and at day 3 or 21, mice were sacrificed and serum and myocardial tissues were harvested. For protein-based analyses, including 4D proteomics and Western blotting, the left ventricle was rapidly dissected, and the infarct/border-zone region supplied by the LAD was collected. The same anatomical sampling strategy was applied across all groups. Mice were housed in cages (3 per cage) with free access to food and water. Predefined exclusion criteria included perioperative death, unsuccessful AMI modeling, severe postoperative distress, rapid body weight loss, or terminal condition before the planned endpoint. Successful AMI modeling was confirmed by visible blanching of the left ventricular anterior wall after LAD ligation and postoperative impairment of cardiac function. During the experiments, no mice died after surgery, no mice were identified as modeling failures, and no mice were excluded according to the predefined exclusion criteria. Therefore, all animals that underwent randomization were included in the final analyses. Outcome assessments, including echocardiographic measurements, Western blot quantification, ELISA, immunofluorescence analysis, TUNEL-positive cell counting, Sirius red fibrosis quantification, and WGA-based cardiomyocyte size measurement, were performed by investigators blinded to group allocation. Group information was decoded only after completion of data acquisition and quantitative analysis.

### 2.3 Proteomic analysis

4D Label free quantitative proteomics was performed using myocardial samples collected from the left ventricular infarct/border-zone region as described in Section 2.2. Proteins were extracted using the SDT lysis method (4% (w/v) SDS, 100 mM Tris/HCl pH 7.6, 0.1 M DTT), followed by protein quantification using the BCA method. A suitable amount of protein from each sample was subjected to digestion using the Filter-Aided Sample Preparation (FASP) method with trypsin (Wang et al., 2023). The digest peptides of each sample were desalted on C18 Cartridges, concentrated using vacuum centrifugation, and reconstituted in 40 μL of 0.1% (v/v) formic acid. Liquid Chromatography-Massspectrometry/Mass Spectrometry (LC-MS/MS) analysis was conducted using the timsTOF Pro mass spectrometer (Bruker, United States), coupled with the NanoElute system (Bruker Daltonics), for a duration of 60 min. Peptides were loaded onto a reverse-phase trap column connected to a C18-reversed-phase analytical column in 0.1% formic acid, and separated at a linear gradient of buffer solution (84% acetonitrile and 0.1% formic acid) at a flow rate of 300 nL/min controlled by IntelliFlow technology. The mass spectrometer was operated in positive ion mode, recording ion mobility MS spectra within the mass range of m/z 100–1700 and 1/k0 values between 0.6 and 1.6. Following this, 10 cycles of PASEF MS/MS were performed with a target intensity of 1.5 k and a threshold of 2500 (Wang et al., 2023). The raw MS data were combined and searched using MaxQuant 1.5.3.17 software for identification and quantitation analysis.

### 2.4 Echocardiography

Transthoracic echocardiography was performed following surgery using the VisualSonics Vevo 2100 imaging system (FUJIFILM, Canada). Briefly, the mice were anesthetized with 2–3% isoflurane and maintained with 1–1.5% isoflurane. Left ventricular (LV) ejection fraction (EF) were measured from the short-axis M-mode at mid-ventricular level with heart rates remaining 450–550 beats per minute (bpm). All parameters were measured three times, and means were used for analysis (Lai et al., 2024). At the end of the procedures all mice recovered from anesthesia without complication.

### 2.5 Histological analysis

Hearts were fixed in 4% paraformaldehyde for 48 h and imbedded in paraffin. Then, 5-μm slides were prepared. Hematoxylin and eosin (H&E) staining was performed for general histological observation, while myocardial fibrosis was mainly evaluated by Sirius red staining. Sirius red stain kit was used to quantify the fibrosis area in the left ventricle (LV) with ImageJ software. The fibrosis rate was determined by dividing the size of the fibrosis region (red area) by the total LV area (Pan et al., 2023). The TUNEL kit was used as directed by the manufacturer to examine apoptotic cardiomyocytes in the infarcted heart. The sections were incubated with the TUNEL reaction mixture for 1 h at room temperature before being stained with DAPI (Lai et al., 2024). The proportion of apoptotic cells (TUNEL+)/total cells were calculated. For immunofluorescence staining, paraffin embedded tissue sections of hearts from different group were deparaffinized, rehydrated and permeabilized by PBS supplemented with 0.1% TritonX-100 for 10 min, blocked by PBS supplemented with 5% BSA for 1 h at room temperature, and then incubated with primary antibodies overnight at 4 °C and the secondary antibodies for 1 h at room temperature. The nuclei were stained with DAPI after washing. For WGA staining, paraffin-embedded heart sections were deparaffinized, rehydrated, and incubated with fluorescently conjugated wheat germ agglutinin at 10 μg/mL for 45 min at room temperature in the dark to delineate cardiomyocyte membranes, followed by nuclear counterstaining with DAPI. Images were acquired using a Pannoramic Scanner (3DHISTECH, Hungary). Myocardial cross-sectional area was quantified using ImageJ software based on WGA-positive cardiomyocyte borders. Randomly selected fields from each mouse were analyzed by investigators blinded to group allocation.

### 2.6 WB and ELISA

WB was conducted as reported previously using myocardial samples collected from the left ventricular infarct/border-zone region as described in Section 2.2. Briefly, protein was extracted using RIPA lysis and the concentrations were quantified by BCA protein assay. Proteins (20–40 μg) were loaded with loading buffer and separated on a 4%–12% SDS-PAGE gel. After electrophoresis, the separated molecules are transferred onto polyvinylidene difluoride (PVDF) membrane. After protein transfer, the membrane was blocked with PBS containing 5% skim milk. Then, the membrane was incubated with specific primary antibodies at 4 °C overnight, washed three times and subsequently incubated with secondary antibodies at room temperature for 1 h. Signals were detected by Tanon 5800 Multi Imaging System (Tanon, China). The densitometry of target bands was normalized to internal control GAPDH. Serum was collected and the concentration of C3a was measured using ELISA kit according to the manufacturer’s instructions. Absorption at 450 nm was used.

### 2.7 Molecular docking

The bioactive components of TXL are listed as previously reported (Li et al., 2020). Briefly, TCM systems pharmacology database (TCMSP, https://tcmspw.com/tcmsp.php) and TCM integrative database (TCMID, https://www.megabionet.org/tcmid/) were used to identify candidates by comprehensively searching the principal components of TXL. A total of 112 Active compounds were screened out on the basis of absorption, distribution, metabolism, and excretion (ADME) protocols, with criteria of oral bioavailability ≥30% and drug-likeness ≥0.18.

For molecular docking, the protein crystal structures of C1qa and the proteomics-identified C1s1 database entry were downloaded from the PDB database, and the 3D structure of the small molecules was constructed using Chem3D 20.0, followed by energy minimization under the MMFF94 force field. In this study, molecular docking was performed using AutoDock Vina 1.1.2 software (Eberhardt et al., 2021). Before docking, the receptor protein was prepared using PyMol 2.5.5, which involved removing water molecules, salt ions, and small molecules (DeLano, 2002). The docking box was then set to enclose the entire protein structure. Additionally, ADFRsuite 1.0 was used to convert the prepared small molecules and receptor protein into the PDBQT format required for AutoDock Vina 1.1.2 docking (Ravindranath et al., 2015). During docking, the global search detail was set to 32, while the remaining parameters were kept at their default settings. The highest-scoring docking conformation was considered the binding conformation. Finally, the docking results were visualized and analyzed using PyMol 2.5.5 and Discovery Studio Visualizer.

### 2.8 Statistical analysis

For conventional phenotypic measurements, data were expressed as the mean ± standard deviation (SD), and comparisons among groups were evaluated by one-way ANOVA followed by Tukey’s *post hoc* test using GraphPad Prism 9.0. For proteomic profiling, differential protein expression was assessed across all quantified proteins, and multiple-testing correction was performed using the Benjamini–Hochberg false discovery rate (FDR) procedure. Both nominal *P*-values and FDR-adjusted q-values were reported at the protein level. Proteins were considered significantly differentially expressed when they met the criteria of q < 0.05 and fold change >1.5 or <2/3. KEGG pathway enrichment analysis was corrected for multiple testing, and adjusted *P*-values/q-values were reported for enriched pathways. An adjusted *P*-value or q-value <0.05 was considered statistically significant for proteomic and pathway enrichment analyses.

## 3 Results

### 3.1 Proteomic results indicate complement cascades are significantly hampered by TXL treatment

**FIGURE 1:**
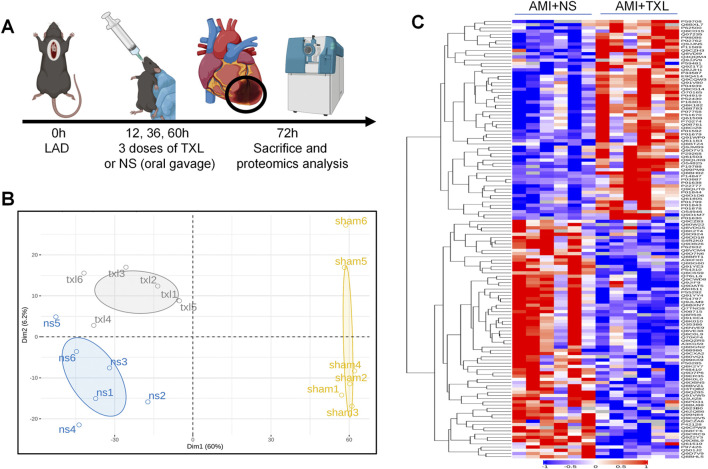
The overview of the proteomic results of mice treated with NS or TXL. n = 6 mice per group for proteomic analysis. **(A)** The scheme of the proteomic analysis. **(B)** The PCA of proteomic results. **(C)** The heatmap of differentially expressed proteins (FC > 1.5 or <2/3, *p* < 0.05) between the AMI + NS and the AMI-TXL group.

**FIGURE 2:**
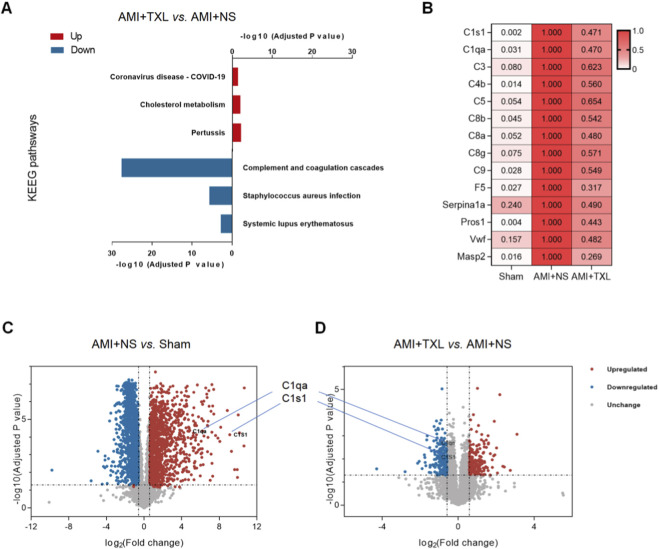
Activation of complement cascades is significantly hampered by TXL treatment. **(A)** KEGG pathway enrichment analysis was corrected for multiple testing using the Benjamini–Hochberg method, and adjusted P-values are shown. **(B)** The expression of several key proteins associated with complement and coagulation cascades. C1s1 refers to the database-annotated protein entry identified in the proteomic analysis. The numbers in cells showed the averaged expression levels. **(C,D)** The volcano plots of differentially expressed proteins between the AMI + NS and the Sham groups **(C)** and between the AMI + TXL and the AMI + NS groups **(D)**.

After 3-dose of TXL or NS, hearts were harvested and protein was extracted, followed by proteomic analysis (Figure 1A). Principal component analysis (PCA) revealed significant intergroup separation of metabolites in the Sham group, the AMI + NS group and the AMI + TXL group (Figure 1B). In a total of 4,640 detectable proteins, 998 proteins showed statistically significant differences between the NS and TXL groups, among which 420 had a fold change >1.5 or <2/3 (Figure 1C). Complete nominal P-values and FDR-adjusted q-values are provided in Supplementary Tables 3,4. KEGG enrichment analysis was performed with multiple-testing correction. Complement and coagulation cascades remained significantly enriched among TXL-regulated proteins after Benjamini–Hochberg correction adjusted indicating that TXL treatment was associated with suppression of complement- and coagulation-related pathways (Figure 2A). The complete KEGG enrichment results, including nominal P-values, and q-values, are provided in Supplementary Table 5. A series of associated components were listed in Figure 2B. Considering that almost all key components of the complement system, including C1qa and C1s, were downregulated, and that the C1s-related protein identified in the proteomic dataset was annotated as C1s1, we hypothesized that this was due to the inhibition of its activation initiation phase by TXL, thereby alleviating the downstream cascade. The volcano plot also confirmed that C1s, annotated as C1s1 in the proteomic dataset, and C1qa were significantly upregulated after myocardial infarction, whereas TXL markedly reduced their levels (Figures 2C,D). These findings support that TXL significantly mitigates the activation of C1-initiated complement activation.

### 3.2 TXL may modulate C1s/C1qa-associated complement activation

**FIGURE 3:**
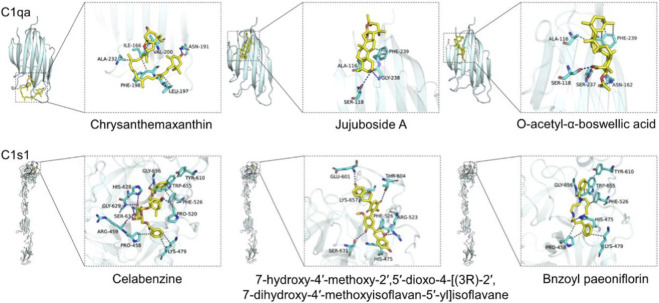
Representative predicted docking poses between of C1qa or the proteomics-identified C1s1 database entry with TXL-derived compounds. The left image shows the overall view, while the right image shows the partial view. The yellow sticks represent small molecules, the cyan cartoon represents the protein, the blue lines indicate hydrogen bonding interactions, and the gray dashed lines represent hydrophobic interactions.

Considering the complex composition of TXL, the potential inhibitory mechanisms of its constituent components on C1s and C1qa were explored, based on the proteomics-identified C1s1 database entry and subsequent C1s protein validation. Molecular docking analysis predicted that multiple bioactive compounds such as jujuboside, chrysanthemaxanthin, O-acetyl-α-boswellic acid, 7-hydroxy-4′-methoxy-2′,5′-dioxo-4-[(3R)-2′,7-dihydroxy-4′-methoxyisoflavan-5′-yl]isoflavane, celabenzine, and bnzoyl paeoniflorin, may potentially interact with C1qa or the proteomics-identified C1s1 database entry. (Table 1; Figure 3). Although the C1s-related protein detected in the proteomic dataset was annotated as C1s1, C1s2 was not reliably detected across samples. Therefore, subsequent antibody-based validation focused on C1s protein expression.

**TABLE 1:** Molecular docking results of the active components in TXL with C1s1* and C1qa.

Molecule ID	Docking Score (kcal/mol)	Molecule name	Molecule ID	Docking Score (kcal/mol)	Molecule name
C1qa	​	​	C1s1	​	​
MOL001527	−7.795	Jujuboside A_qt	MOL002967	−10.206	7-Hydroxy-4′-methoxy-2′,5′-dioxo-4-[(3 R)-2′,7-dihydroxy-4′-methoxyisoflavan-5′-yl]isoflavane
MOL004492	−7.53	Chrysanthemaxanthin	MOL005314	−9.723	Celabenzine
MOL001241	−7.269	O-acetyl-α-boswellic acid	MOL007003	−9.261	Bnzoyl paeoniflorin
MOL001265	−7.231	Acetyl-alpha-boswellic,acid	MOL001921	−9.25	Lactiflorin
MOL007003	−7.201	Bnzoyl paeoniflorin	MOL002776	−9.165	Baicalin
MOL005376	−7.197	Panaxadiol	MOL001243	−9.084	3alpha-hydroxy-olean-12-en- 24-oic-acid
MOL001921	−7.065	Lactiflorin	MOL001527	−8.987	Jujuboside A_qt
MOL005043	−7.047	Campest-5-en-3beta-ol	MOL001924	−8.851	Paeoniflorin
MOL000211	−7.045	Mairin	MOL007025	−8.842	Isobenzoylpaeoniflorin
MOL006865	−7.037	Dipterocarpol	MOL001265	−8.76	Acetyl-alpha-boswellic,acid
MOL002967	−7.035	7-Hydroxy-4′-methoxy-2′,5′-dioxo-4-[(3 R)-2′,7-dihydroxy-4′-methoxyisoflavan-5′-yl]isoflavane	MOL001255	−8.726	Boswellic acid
MOL007025	−6.925	Isobenzoylpaeoniflorin	MOL004492	−8.576	Chrysanthemaxanthin
MOL001243	−6.872	3alpha-hydroxy-olean-12-en- 24-oic-acid	MOL000787	−8.532	Fumarine
MOL005401	−6.823	Ginsenoside Rg5_qt	MOL000354	−8.478	Isorhamnetin
MOL001255	−6.804	Boswellic acid	MOL001539	−8.46	Sanjoinenine
MOL000787	−6.751	Fumarine	MOL005348	−8.439	Ginsenoside-Rh4_qt
MOL001542	−6.667	Swertisin	MOL001525	−8.391	Daucosterol
MOL006999	−6.631	Stigmast-7-en-3-ol	MOL004355	−8.356	Spinasterol
MOL000358	−6.625	Beta-sitosterol	MOL003000	−8.347	Stevein
MOL006861	−6.606	Asiatic acid	MOL002982	−8.333	(3 R,4 R)-3′,7-dihydroxy-2′,4′-dimethoxy-4-[(2 S)-4′,5,7-trihydroxyflavanone-6-yl]isoflavan
MOL000449	−6.598	Stigmasterol	MOL002975	−8.332	Butin
MOL001539	−6.531	Sanjoinenine	MOL005376	−8.293	Panaxadiol
MOL001215	−6.514	Tirucallol	MOL001522	−8.268	(S)-Coclaurine
MOL002322	−6.477	Isovitexin	MOL005399	−8.251	Alexandrin_qt
MOL001263	−6.476	3-Oxo-tirucallic,acid	MOL005384	−8.179	Suchilactone
MOL007004	−6.473	Albiflorin	MOL000449	−8.127	Stigmasterol
MOL002776	−6.419	Baicalin	MOL000006	−8.111	Luteolin
MOL005314	−6.378	Celabenzine	MOL001542	−8.094	Swertisin
MOL005356	−6.355	Girinimbin	MOL005401	−8.093	Ginsenoside Rg5_qt
MOL000359	−6.35	Sitosterol	MOL002940	−8.038	(3 R)-3-(2,3-dihydroxy-4-methoxyphenyl) -7-hydroxychroman-4-one

* C1s1 refers to the database-annotated protein entry used for proteomic identification and molecular docking. Subsequent antibody-based validation was performed at the C1s protein level.

**FIGURE 4:**
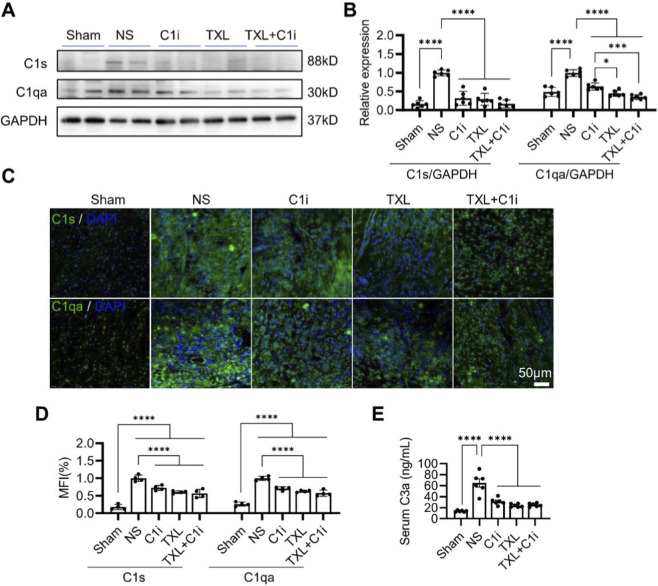
Adding C1i did not further enhance the complement inhibition and cell-protective effects of TXL. n = 6 mice per group for WB and ELISA analyses; n = 4 mice per group for immunofluorescence. **(A)** The WB results of C1s and C1qa. **(B)** The quantitative analysis of C1s and C1qa. **(C)** The representative images of immunofluorescent staining of C1s and C1qa. Bar = 50 μm. **(D)** The quantitative analysis of immunofluorescent staining. **(E)** The serum C3a levels. *p < 0.05; **p < 0.01; ***p < 0.001; ****p < 0.0001.

To further investigate whether TXL could inhibit C1s and C1qa in AMI models, TXL or C1i was administered separately or in combination. C1s and C1qa levels in heart tissue were downregulated by WB and immunofluorescent staining in all three groups received treatment compared with the NS-treated group (Figures 4A–D). Consistently, the concentration of C3a in serum was lower in the three groups treated with C1i, TXL or the combination (Figure 4E). Although the effect of TXL is significantly enhanced or shows a trend of enhancement compared to C1i, combination therapy failed to demonstrate additional benefit over TXL alone. These data suggest that C1-associated complement activation may be involved in the cardioprotective effects of TXL.

### 3.3 C1-mediated component cascades activation serves as one of the mechanisms underlying the cardioprotective effects of TXL

**FIGURE 5:**
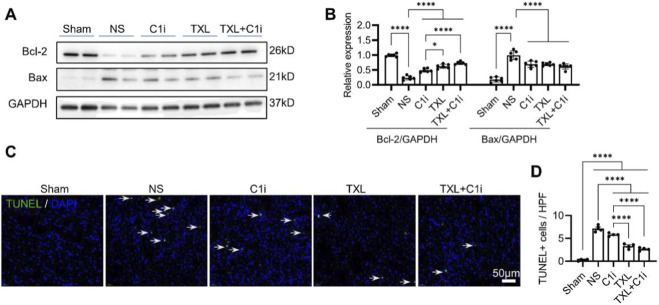
C1-mediated complement cascades is one of the mechanisms underlying the protective effects of TXL against cell death. **(A)** The WB results of Bcl-2 and Bax, two essential survival-related proteins (n = 6). **(B)** The quantitative analysis of Bcl-2 and Bax. **(C)** The representative images of TUNEL staining (n = 4). Bar = 50 μm. **(D)** The quantitative analysis of TUNEL+ cells per HPF. *p < 0.05; **p < 0.01; ***p < 0.001; ****p < 0.0001.

**FIGURE 6:**
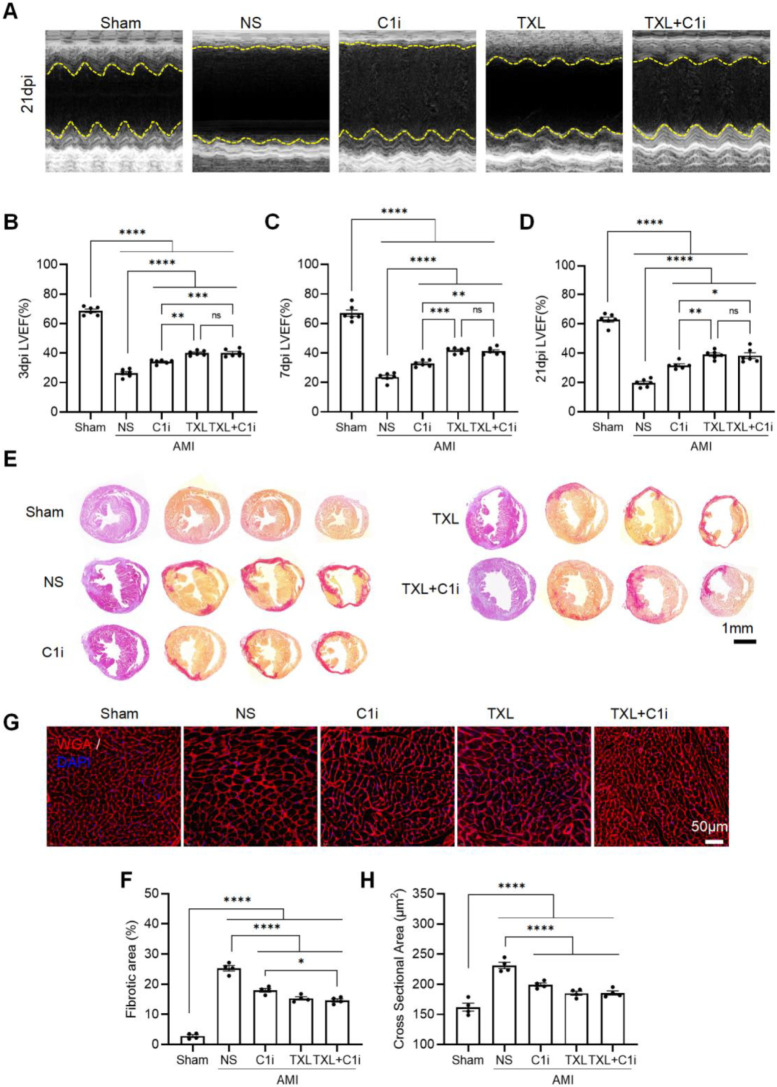
C1-mediated complement cascades is one of the mechanisms underlying the cardioprotective effects of TXL. n = 6 mice per group for echocardiography, Sirius red staining, and WGA staining. **(A)** The representative images of echocardiography. **(B–D)** The quantitative analysis of LVEF at day 3 **(B)**, 7 **(C)**, and 21 days **(D)** after AMI. **(E)** The representative images of Sirius red staining. Bar = 1 mm. **(F)** The quantitative analysis of Sirius red staining. Fibrotic area is identified as red area. **(G)** The representative images of WGA staining. Bar = 50 μm. **(H)** The quantitative analysis of WGA staining. *p < 0.05; **p < 0.01; ***p < 0.001; ****p < 0.0001.

Given that complement cascades contribute to cell death and tissue injury, we investigated the expression levels of related proteins, Bcl-2 and Bax (Figures 5A,B). AMI Mice had a significantly upregulation of Bax (a pro-apoptotic protein) and a downregulation of Bcl-2 (a pro-survival protein). Both C1i and TXL promoted survival and alleviated cell death. Consistently, cell death was mitigated by TXL and C1i treatment in terms of TUNEL staining (Figures 5C,D). Cardiac performance was also improved at day 3, 7 and 21 post-AMI in the C1i, TXL, or combined therapy in terms of left ventricular systolic function measured by echocardiography (Figures 6A–D). TXL-treated mice had a moderately significant improvement in LVEF compared with those C1i-treated mice; while addition of C1i to TXL did not further improve cardiac ejection function. Moreover, evaluation of fibrotic area (Figures 6E,F) and cardiomyocyte hypertrophy (Figures 6G,H) confirmed that TXL attenuated cardiac remodeling. These findings support that TXL may protect against myocardial infarction injury through multiple mechanisms, including inhibition of C1-mediated complement activation and other pathways. This is because TXL alone appears to be more effective than C1 inhibition alone, and combination therapy does not offer superior benefits over TXL monotherapy.

## 4 Discussion

In this study, proteomics and experimental analyses suggest that modulation of C1-associated complement activation is one of the mechanisms underlying the cardioprotective effect of TXL in myocardial infarction. This provides new mechanistic insights into the therapeutic potential of this promising traditional Chinese medicine in the treatment of myocardial ischemia.

The complement system is a complex network of membrane-associated and cell surface proteins that plays a critical role at the interface of innate and adaptive immunity. Subcellular substances released from ischemic cardiomyocytes, such as cardiolipin, can activate the complement cascade, leading to C3 cleavage, followed by C5 activation and the formation of the membrane attack complex. Cardioprotective effects of several inhibitors targeting complement cascades have been explored, such as CVF, FUT-175 (also known as nafamostat mesylate), blocking antibodies, soluble CR1 (sCR1) and C1i (also known as Serpin G1). CVF and recombinant humanized CVF, which deplete complement factor C3, have been shown to reduce ischemia/reperfusion (I/R) injury in rats (Gorsuch et al., 2009). Similarly, FUT-175 is a synthetic protease inhibitor that suppresses complement activation by binding to C1r and C1s, thereby preventing C1-mediated cleavage of C2 and C4. In a rabbit model of myocardial ischemia-reperfusion, Schwertz et al. administered FUT-175 and found that it reduced infarct size, inflammatory infiltration, and plasma creatine kinase (CK) levels. Monoclonal antibodies targeting C5, C5a, and the C5a receptor have been shown to reduce myocardial infarction size in rodent and porcine models (Van der Pals et al., 2010; Timmers et al., 2012). Recombinant human sCR1 and C1i are potent complement inhibitors that reduce infarct size in various animal models (Weisman et al., 1990; Buerke et al., 1998). CR1 can inhibit both the classical and alternative pathways by dissociating the C3 and C5 convertases and acts as a cofactor for the factor I-mediated degradation of C3b and C4b, while C1i is a plasma protein that covalently counteracts the assembly of C1 and the subsequent activation of the classical complement pathway. In addition to its inhibitory effects on the complement system, C1i also modulates the coagulation system and has been reported to suppress the adhesion of leukocytes to endothelial cells (Panagio et al., 2018). Only a few complement inhibitors have been approved by the U.S. Food and Drug Administration (FDA) for clinical use, including C1-inhibitor (Rother et al., 2007). Fattouch et al. intravenously administered C1i (500 U) as a bolus during surgery, followed by a 3-h infusion of 500 U postoperatively. They found that, 2 hours after surgery, cardiac function improved, and both intubation and hospitalization times were shortened. However, C1i did not significantly affect mortality, bleeding, renal failure, or complications such as arrhythmias (Fattouch et al., 2007). Regarding the C5 complement inhibitor Pexelizumab, a Phase II trial in patients with ST-elevation myocardial infarction undergoing percutaneous coronary intervention (PCI) showed no significant effect on infarct size but a significant reduction in 90-day mortality (Granger et al., 2003). In summary, C1i or other complement-based interventions could exert a protective effect following myocardial infarction, but efforts over the past 2 decades focusing on it as a sole therapeutic target appeared to have demonstrated limited efficacy. Nevertheless, as one of the multiple protective mechanisms of other potential therapies, it remains an important factor that should not be overlooked. Herein, a 3-dose therapy of TXL or C1i was adopted since Wysoczynski et al. found that MAC levels were the highest 48 h after AMI modeling, which differed only minimally from controls after 72 h (Wysoczynski et al., 2014). We found that both TXL and C1i could inhibit the activation and accumulation of C1 within tissues, with TXL exhibiting a stronger effect on C1qa than C1i (0.4 IU/g body weight). In monotherapy, TXL outperformed C1i in suppressing cell death and improving cardiac function, likely due to the involvement of additional mechanisms. Combination therapy did not further enhance the protective effect of TXL, suggesting that C1 inhibition is one of the key inherent effects of TXL.

TXL is a compound formulation composed of 12 natural ingredients, including ginseng, leech, scorpion, and red peony, and has been approved in China for the treatment of angina pectoris and ischemic stroke for nearly 30 years (Zhang et al., 2019). In a small-scale study (CAPITAL), TXL capsules were shown to stabilize arterial plaques, reduce major cardiovascular events, and delay the time to the first event (Zhang et al., 2019). In a large, randomized, double-blind, placebo-controlled, multicenter clinical trial (CTS-AMI) aimed at evaluating the efficacy and safety of TXL as an adjunct to guideline-directed therapy (including direct percutaneous coronary intervention) in Chinese community-based STEMI patients, we found that TXL improved clinical outcomes at 30 days and 1 year (Yang et al., 2023). Previous reports suggest that the mechanisms underlying the cardiovascular benefits of TXL are complex and involve regulating activity of nitric oxide synthase (Chen et al., 2020; Cheng et al., 2009), inhibiting ferroptosis and pyroptosis (Wang et al., 2022; Jiang et al., 2023), and enhancing autophagy (Chen et al., 2021). Yet, there has been no proteomic analysis of the myocardium to aid in understanding the mechanisms of TXL in the treatment of AMI from a comprehensive perspective. Herein, complement and coagulation cascades were identified as one of the pathways associated with the cardioprotective effects of TXL, and C1s/C1qa may represent important complement-related proteins affected by TXL treatment. A total of 111 identifiable chemical ingredients were collected within TXL prescription from TCM systems pharmacology (TCMSP) and TCM integrative database (TCMID) databases, among which a series of candidates were predicted to block C1qa or C1s, as shown in the molecular docking analysis (Li et al., 2020). More efforts are required to identify the specific ingredient exerting the anti-complement effects.

## 5 Limitations

Several limitations should be noted. First, the *in vivo* AMI model in this study was established by permanent LAD ligation without reperfusion. Although this model is widely used to investigate post-infarction myocardial injury, complement activation, inflammation, and cardiac remodeling, it does not fully reproduce the pathophysiological conditions of reperfused AMI after primary PCI. Thus, future studies using ischemia-reperfusion injury models are warranted to further determine whether the complement-regulatory and cardioprotective effects of TXL are preserved under reperfusion conditions. In addition to this limitation in model selection, the target-binding evidence also requires further validation. Although molecular docking suggested potential interactions between several TXL-derived compounds and C1qa or the proteomics-identified C1s1 database entry, these findings should be interpreted as computational predictions rather than evidence of direct physical binding or functional inhibition. In particular, C1qa is not a typical enzymatic target, and the use of a full-protein docking box may generate non-specific binding poses. Further biophysical validation, such as surface plasmon resonance, microscale thermophoresis, isothermal titration calorimetry, cellular thermal shift assay, or thermal shift assays, is required to confirm direct ligand–target binding. Moreover, we did not perform C1s enzymatic activity assays or functional assays of the classical complement pathway; therefore, whether the predicted TXL-derived compounds directly inhibit C1s activity or classical complement activation remains to be determined. Finally, the myocardial concentrations of the predicted TXL-derived compounds after intragastric administration were not measured in this study. Thus, whether these compounds can reach pharmacologically effective concentrations in myocardial tissue requires further pharmacokinetic and tissue-distribution studies.

## 6 Conclusion

Conclusively, inhibition of C1-mediated complement cascades is considered a part of mechanisms of the cardioprotection of TXL, which leads to reduced cell death and tissue injury. These data could contribute to understand the benefits of the well-recognized traditional Chinese medicine.
